# Pediatric community-acquired pneumonia: predictive value of heparin-binding protein for severity assessment

**DOI:** 10.1038/s41390-025-04605-w

**Published:** 2025-12-11

**Authors:** Nagwan Y. Saleh, Fahima M. Hassan, Thoria A. Omar, Mahmoud F. Al Gady, Rania S. El-Deen Hussein

**Affiliations:** 1 Pediatrics Department, Faculty of Medicine, Menoufia University, Shebin El-kom, Egypt; 2 Clinical Pathology Department, Faculty of Medicine, Menoufia University, Shebin El-kom, Egypt

## Abstract

**Background:**

Heparin-Binding Protein (HBP) is a promising biomarker for predicting the severity of community-acquired pneumonia (CAP). This study aimed to evaluate its role in pediatric CAP severity assessment.

**Methods:**

A prospective observational study was conducted at the Pediatric Intensive Care Unit (PICU)and general wards of Menoufia University Hospital from February 2024 to October 2024. Thirty children with mild pneumonia (Simple Pneumonia) and 60 with severe pneumonia admitted to the PICU (Severe Pneumonia) were enrolled. Severity was assessed using WHO criteria and several clinical scores: Pediatric Respiratory Severity Score(PRESS), Respiratory Index of Severity Score (RISC), modified Predisposition, Insult, Response, and Organ dysfunction(PIROm), Pediatric Risk of Mortality(PRISM), Pediatric Index of Mortality2 (PIM2), and pediatric Sequential Organ Failure Assessment (pSOFA). Serum HBP levels were measured upon admission.

**Results:**

Mean HBP levels were significantly higher in severe pneumonia compared with simple pneumonia [7.87 ng/mL vs 0.73 ng/mL, *p* < 0.001]. All severity scores were also significantly elevated in this group and HBP was significantly correlated with these severity scores. In multivariate logistic regression, HBP was an independent predictor of pneumonia severity (adjusted odd ratio=3.23; 95%confidence interval: 1.72–6.06; *p* < 0.001).HBP showed excellent predictive performance, with an area under the curve (AUC) of 0.962 for severe pneumonia. A cut-off >1.2 ng/mL for HBP yielded 100% sensitivity and 90% specificity.

**Conclusions:**

Serum HBP is a reliable and highly accurate biomarker for assessing CAP severity in pediatric patients and may support early clinical decision-making.

**Impact statement:**

- **Early Risk Stratification**: This study identifies Heparin-Binding Protein (HBP) as a powerful biomarker for early prediction of disease severity in pediatric community-acquired pneumonia, with an excellent diagnostic performance (AUC 0.962).
- **Clinical Utility**: HBP demonstrated 100% sensitivity and 90% specificity at a cut-off >1.2 ng/mL, outperforming traditional clinical scores in distinguishing severe from mild cases, which can aid timely PICU admission decisions.
- **Potential for Practice Integration**: Incorporating HBP measurement into initial assessment protocols may enhance clinical decision-making, improve outcomes, and optimize resource allocation, especially in settings with high pediatric pneumonia burden.
- **Cost and Availability of Heparin-Binding Protein**: HBP levels were measured using a commercially available ELISA kit, as the test is not yet widely available in routine clinical laboratories. The assay cost ranges from approximately $400 to $800 per 96-well plate, with an estimated per-sample cost of $10–30. Due to the limited availability of clinical-grade HBP assays, testing was conducted under research conditions in a controlled laboratory setting.

## Introduction

Community-acquired pneumonia (CAP) is a heterogeneous disease, ranging from mild, self-limited illness to severe infections that can lead to respiratory failure, septic shock, and even death.^1^ Studies from various populations report that the incidence of severe CAP among hospitalized children ranges from 3.9% to 23.0%.^2–4^

At present, no single biomarker has been established as the definitive marker for assessing CAP severity. There remains a need for rapid and effective biomarkers to overcome the limitations of traditional indicators and aid in treatment decision-making. One such novel biomarker is Heparin-Binding Protein (HBP), a member of the serine protease family. Stored in the secretory and azurophilic granules of neutrophils, HBP is rapidly released upon neutrophil stimulation during inflammatory responses.^5^

HBP has been proposed as a potential biomarker for sepsis progression, given its ability to induce vascular leakage and modulate various cellular responses during inflammation.^6^ Its prognostic value for predicting sepsis severity has been well-established in adult populations.^7^ Recent systematic reviews and meta-analyses have evaluated the diagnostic and prognostic value of HBP in sepsis and other severe infections. Pooled data indicate that HBP demonstrates moderate-to-high diagnostic accuracy, sometimes outperforming conventional biomarkers such as C-reactive protein and, in specific contexts, procalcitonin. However, substantial heterogeneity and the limited availability of pediatric-specific data highlight the need for further prospective studies and standardized assay protocols before routine clinical adoption.^7–9^

Additionally, HBP has been shown to have diagnostic and discriminative value in the etiology of CAP, with studies indicating that HBP offers superior predictive ability for distinguishing between bacterial and viral infections compared to traditional markers such as leukocyte and neutrophil counts.^10^

Huang et al. evaluated the role of HBP in critically ill children with severe CAP and reported that HBP levels were significantly elevated in more severe cases. Their findings suggested that HBP might outperform conventional biomarkers in predicting disease progression, proposing it as a promising tool for early risk stratification in this population. These results support further investigation into the diagnostic and prognostic value of HBP in pediatric CAP.^11^

Further supporting its clinical relevance, Huang et al. demonstrated that HBP levels in bronchoalveolar lavage fluid could effectively discriminate between severe bacterial and viral pneumonia in critically ill children, offering insights into etiological diagnosis and therapeutic decisions.^12^ Additionally, Li et al. reported that elevated HBP levels were significantly associated with severe or complicated CAP in pediatric populations, further validating HBP’s role as a prognostic biomarker in this context.^13^ Given these findings, the objective of our study was to assess the role of HBP in predicting the severity of CAP in pediatric patients.

## Patients and methods

### Patients

This study was conducted on two groups of children diagnosed with community-acquired pneumonia (CAP). Group 1 (Simple Pneumonia) included children admitted to the general pediatric wards, while Group 2 (Severe Pneumonia) consisted of children admitted to the Pediatric Intensive Care Unit (PICU). All participants were admitted to the Pediatric Department at Menoufia University Hospital, Egypt, between February 2024 and October 2024. Written informed consent was obtained from the parents or guardians of all participants. The study protocol was approved by the Ethics Committee of Menoufia University.

**Inclusion criteria** were as follows: 1- Children aged between 2 months and 59 months, based on the revised 2014 World Health Organization (WHO) guidelines, 2- Informed parental consent and 3- Clinical and radiological diagnosis of community-acquired pneumonia (CAP), defined as a lung infection acquired outside of a healthcare setting in previously healthy children, confirmed by the presence of pulmonary consolidation on chest radiography in accordance with the British Thoracic Society guidelines.^14^

Children were excluded if they were younger than 2 months or older than 59 months; had co-existing infections (e.g., gastroenteritis); had suspected or confirmed tuberculosis; had known immunodeficiency or were receiving current chemotherapy; had chronic pulmonary or cardiac disease; had liver or renal dysfunction; had a history of recent antibiotic use or hospitalization; or if consent was refused.

Patients were classified based on the revised World Health Organization (WHO) 2014 guidelines^15^ into **Simple pneumonia** was diagnosed in the presence of tachypnea and/or chest indrawing or **Severe pneumonia** was diagnosed in the presence of one or more general danger signs, including persistent vomiting, lethargy or unconsciousness, seizures, severe malnutrition, stridor when calm, or inability to drink. Patients were admitted to the PICU if they fulfilled any of the following criteria: (1) requirement for invasive or noninvasive mechanical ventilation, (2) evidence of impending respiratory failure, (3) oxygen saturation (SpO₂) <92% despite an inspired oxygen concentration ≥50%, (4) clinical signs of shock, or (5) altered mental status.^16^ All other patients were managed in general ward settings at the discretion of the treating physician.

### Severity and outcome scoring systems

Three pneumonia-specific severity scores were utilized in this study: the Pediatric Respiratory Severity Score (PRESS), the Respiratory Index of Severity Score (RISC), and Predisposition, Insult, Response, and Organ dysfunction modified score (PIROm). **PRESS** is a CAP-specific tool that quantifies respiratory distress and oxygen requirement and was validated through a prospective study in a Japanese pediatric population. A score of 4–5 is indicative of severe pneumonia.^17^ **RISC** is designed to predict mortality risk in pediatric pneumonia, particularly in low-resource settings, using clinical signs and oxygen saturation and was developed and validated in South African pediatric populations, where a score of ≥3 suggests a poor prognosis.^18^ **PIROm** stratifies pneumonia severity by combining patient risk factors, infection characteristics, host inflammatory response, and degree of organ dysfunction and was validated in a retrospective study conducted in Paraguay. Scores of 5–6 denote severe pneumonia, while scores between 7–10 indicate very severe pneumonia.^19^

In addition to disease severity scores, we employed three systems to assess mortality risk and sepsis severity: **Pediatric Risk of Mortality (PRISM) score** predicts ICU mortality and calculated within the first 24 hours of admission using 14 clinical and laboratory variables. These values were input into the official PRISM calculator (http://www.sfar.org/scores2/prism2.php), which provides an estimated mortality risk.^20^ **Pediatric Index of Mortality 2 (PIM2)** is a validated ICU mortality prediction model calculated at admission, useful for benchmarking and risk adjustment and is a faster scoring tool applied within one hour of the patient’s initial clinical evaluation. This score correlates with predicted mortality.^21^ **pediatric Sequential Organ Failure Assessment (pSOFA)** score, evaluates the extent of multi-organ dysfunction and is widely used to define and grade pediatric sepsis severity and used to evaluate the degree of organ dysfunction. A pSOFA score of ≥2 is associated with a 2- to 25-fold increase in mortality risk compared to patients with scores <2.^22^

The *primary outcome* of the study was to evaluate the utility of Heparin-Binding Protein as a biomarker in predicting pneumonia severity. *Secondary outcomes* included hospital length of stay and correlations between serum HBP levels and the clinical, laboratory, and scoring parameters among children diagnosed with CAP.

### Methodology

A comprehensive clinical evaluation was performed for all patients, including detailed history taking and physical examinations. Vital signs and oxygen saturation levels were continuously monitored. Hypoxia was defined as a sustained peripheral SPO₂ below 94%.^23^ Shock was defined as a clinical syndrome of circulatory dysfunction. This includes inadequate tissue perfusion and oxygen delivery that may present with signs such as altered mental status, delayed capillary refill ( > 2 seconds), tachycardia, weak peripheral pulses, cool extremities, hypotension (late sign), decreased urine output, and metabolic acidosis.^24^

Blood samples for Heparin-Binding Protein (HBP) detection were collected immediately upon hospital admission, whether to the Pediatric Intensive Care Unit (PICU) or the general pediatric ward, prior to any escalation of care. Samples were drawn into sodium citrate tubes to prevent clotting and were processed within 30 minutes. Plasma was separated by centrifugation and analyzed for HBP levels within two hours of collection. Serum HBP concentrations were measured using a commercial human azurocidin-1 (AZU1) sandwich ELISA kit (Cloud-Clone Corp., Houston, TX, Catalog No. SEA528Hu; Lot No. XXXX). Human azurocidin-1 (AZU1) is synonymous with HBP. The assay detection range was 0.156–10 ng/mL. All procedures were performed strictly according to the manufacturer’s instructions. Serum samples were diluted 1:50 prior to measurement. Samples with optical density values exceeding the upper limit of the standard curve were re-analyzed after further dilution (up to 1:200), and the final concentrations were calculated after correcting for the dilution factor. According to the manufacturer’s validation data, the assay demonstrates spike-recovery of 91–107% and dilution linearity with R² >0.99 across serial dilutions. In our study, all standard curves generated achieved acceptable curve fitting (R² >0.98). The sensitivity, or lower limit of detection (LLD), is <0.051 ng/mL, which represents the lowest concentration that can be statistically distinguished from zero (calculated as the mean optical density of twenty zero-standard replicates + 2 SD). The assay showed good reproducibility, with intra-assay CV < 10% and inter-assay CV < 12%. The assay is intended for research use only and does not specify normal reference values; therefore, HBP concentrations in this study were interpreted based on their distribution in the study population and reference values from relevant published literature. In addition to HBP, other conventional biomarkers such as C-reactive protein (CRP) and white blood cell counts (WBCs) were also measured and recorded for comprehensive analysis.

### Sample size calculation

The sample size was estimated based on prior findings from Li et al., who reported that, the rate of severe or complicated CAP among patients with upper-quartile HBP concentrations was 54.8%, compared to 26.6% among those with lower HBP levels. Using these proportions, the minimum required sample size to detect a statistically significant difference (two-sided test, alpha = 0.05, power = 80% and confidence level is 95) was calculated using the statistics and sample size calculator (version 6). The calculated total sample size was 90 participants, with a 1:2 allocation ratio between simple pneumonia and severe pneumonia groups.^13^

### Statistical analysis

Data analysis was performed using SPSS version 28 (IBM Corp., Armonk, NY). Normality of data distribution was assessed using the Shapiro–Wilk test. Parametric variables are presented as mean ± standard deviation (SD) and compared using the independent- samples t test, while non-parametric variables are presented as median (interquartile range [IQR]) and compared using the Mann–Whitney U test. Categorical variables are expressed as frequency and percentage and compared using the chi-square or Fisher’s exact test as appropriate. A *p*-value < 0.05 (two-tailed) was considered statistically significant. The Spearman correlation coefficient was calculated to explore associations between continuous variables. Multivariate logistic regression analysis was performed to identify independent predictors of severe pneumonia. The dependent variable was the presence of severe pneumonia (yes/no), while independent variables included demographic, clinical, and laboratory parameters found to be significant in univariate analysis (*p* < 0.1). Adjusted odds ratios (AORs) with 95% confidence intervals were calculated to assess the strength of associations. Model performance was evaluated using the Nagelkerke *R*² to estimate the proportion of variance explained and the Hosmer–Lemeshow test to assess goodness-of-fit. Variables were assessed for multicollinearity prior to inclusion to ensure model stability. Diagnostic accuracy was evaluated by calculating sensitivity, specificity, positive predictive value (PPV), and negative predictive value (NPV). Receiver Operating Characteristic (ROC) curve analysis was conducted to evaluate the diagnostic performance of HBP and clinical severity scores in predicting severe pneumonia. The presence of severe pneumonia was used as the binary outcome variable (severe vs. non-severe). The area under the ROC curve (AUC) was calculated for each parameter to assess its discriminative ability. An AUC > 0.5 was considered acceptable, while an AUC approaching 1.0 indicated excellent diagnostic accuracy. Sensitivity and specificity values for each predictive score were calculated based on ROC-optimized cutoff points using the Youden index.

## Results

The study included 90 pediatric patients diagnosed with community-acquired pneumonia (CAP), of whom 30 (33.3%) had simple pneumonia and 60 (66.7%) had severe pneumonia. Baseline demographic and clinical characteristics, recorded at admission, are presented in Table 1. In terms of clinical outcomes, 14 patients (23.3%) in the severe pneumonia group required mechanical ventilation (MV), with a mean duration of 6.1 days. The overall mortality rate was 6.67%. Patients with severe pneumonia had a significantly longer hospital stay, greater need for MV, and longer duration of MV compared to those with simple pneumonia. Although mortality was observed only in the severe pneumonia group, the difference in mortality rates between groups was not statistically significant, likely due to the small number of deaths (*n* = 4, 6.7%).

**Table 1:** Demographic and clinical data of the studied groups

Variables		Simple pneumonia (*N* = 30)	Severe pneumonia (*N* = 60)	Test of sig.	*P* value
Age/months	Median (IQR)	24 (8–59)	24 (6.75–59)	*U* = 795.50	0.373
Sex	Male	18 (60%)	32 (53.33%)	*X*^2^ = 0.360	0.548
	Female	12 (40%)	28 (46.67%)		
Weight /Kg	Mean ± SD	16.53 ± 4.17	10.93 ± 3.69	*t* = 6.498	<0.001*
Height (cm)	Mean ± SD	103.97 ± 13.04	88.77 ± 18.3	*t *= 4.059	<0.001*
BMI (Kg/m^2^)	Mean ± SD	15.54 ± 4.14	13.93 ± 3.19	*t* = 2.037	0.045*
Hospital stays/ days	Mean ± SD	4.7 ± 1.82	11.63 ± 2.78	*t* =7.37	<0.001*
Need for MV		0 (00.00%)	14 (23.33%)	0.001*	
Duration of MV (days)	Mean ± SD	-	6.1 ± 1.79	t =6.48	<0.001*
Mortality		0 (0%)	4 (6.67%)	0.297	

*IQR* interquartile range, *SD* standard deviation, *BMI* body mass index, *MV* mechanical ventilation, *U* Mann–Whitney U test, *t* independent-samples t test, *X*^2^ chi-square test, * statistically significant.

Statistically significant *p*-values are in bold.

Laboratory findings showed that children with severe pneumonia had significantly elevated levels of Heparin-Binding Protein (HBP) (*P* < 0.001). Additionally, they exhibited significantly lower hemoglobin levels (*P* < 0.001) and significantly higher white blood cell (WBC) counts and platelet counts (*P* < 0.001 and *P* = 0.005, respectively) compared to those with simple pneumonia. C-reactive protein (CRP) levels were also markedly higher in the severe group (*P* < 0.001). Pathogenic bacteria were identified in blood cultures from 13 patients (21.7%). The most frequently isolated organism was Streptococcus pneumonia (7 patients), followed by Staphylococcus aureus (3 patients) and Group A β-hemolytic Streptococcus (3 patients). Radiological assessments revealed no significant differences between the two groups; however, patchy pneumonia was more frequently observed than lobar pneumonia in both cohorts (Table 2).

**Table 2:** Laboratory investigations of the studied groups

		Simple pneumonia (*N* = 30)	Severe pneumonia (*N* = 60)	Test of sig.	*P* value
Hb (gm/dL)	Mean ± SD	11.97 ± 1.39	10.22 ± 1.14	*t *= 313.50	<0.001*
Platelets (*10^9^/L)	Mean ± SD	282.33 ± 36.38	371.62 ± 166.89	*t *= 530.50	0.005*
WBCs (*10^9^/L)	Mean ± SD	6.62 ± 1.18	13.25 ± 5.79	*t* = 145.00	<0.001*
CRP (mg/L)	Median (IQR)	1.7 (1.4 - 2.2)	24 (12–48)	*U* = 0.000	<0.001*
ALT (U/L)	Median (IQR)	21(19-23)	22(20-32.25)	*U* = 614.0	0.014*
AST (U/L)	Median (IQR)	20 (18 – 22)	24 (18–34.5)	*U *= 642.50	0.028*
Albumin (g/dL)	Mean ± SD	3.95 ± 0.38	3.61 ± 0.22	*t* = 5.427	<0.001*
Creatinine (mg/dL)	Mean ± SD	0.46 ± 0.05	0.52 ± 0.3	*t* = 1.124	0.264
Urea (mg/dL)	Mean ± SD	12.97 ± 3.18	20.82 ± 8.09	*t* = 5.111	0.028*
Na (mEq/L)	Mean ± SD	138.93 ± 2.16	136.41 ± 3.02	*t* = 4.071	<0.001*
K (mEq/L)	Mean ± SD	4.07 ± 0.36	4.27 ± 0.7	*t* = 1.457	0.149
Ca (mg/dL)	Mean ± SD	9.78 ± 0.45	9.06 ± 0.55	*t* = 6.195	<0.001*
pH	Mean ± SD	7.4 ± 0.03	7.38 ± 0.08	*t* = 1.197	0.234
CO_2_ (mmol/L)	Mean ± SD	35.93 ± 2.02	41.25 ± 13.08	*t *= 2.207	0.030*
HCO_3_ (mEq/L)	Mean ± SD	25.27 ± 2.07	20.43 ± 4.56	*t* = 5.525	<0.001*
HBP(ng/ml)	Mean ± SD	0.73 ± 0.21	7.87 ± 1.04	*t* = 37.060	<0.001*
Blood Culture (N %)	Positive Negative	0 (0%) 30 (100%)	13(21.7%) 47(78.3%)	*X*^2^ = 5.82	0.02*
Radiological finding (N %)	Lobar pneumonia	11(36.67%)	12(20%)	*X*^2^ = 2.92	0.087
	Patchy pneumonia	19(63.33%)	48(80%)		

*IQR* interquartile range, *SD* standard deviation, *Hb* hemoglobin, *WBCs* white blood cells, *CRP* C-reactive protein, *ALT* alanine aminotransferase, *AST* aspartate aminotransferase, *Na* Sodium, K Potassium, *Ca* Calcium, *CO*_2_ carbon Dioxide, *HCO*_3_ bicarbonate, *HBP* Heparin binding protein, *U* Mann–Whitney U test, *t* independent- samples t test, *X*^2^ chi-square test, * statistically significant.

Statistically significant *p*-values are in bold.

**Fig. 1:**
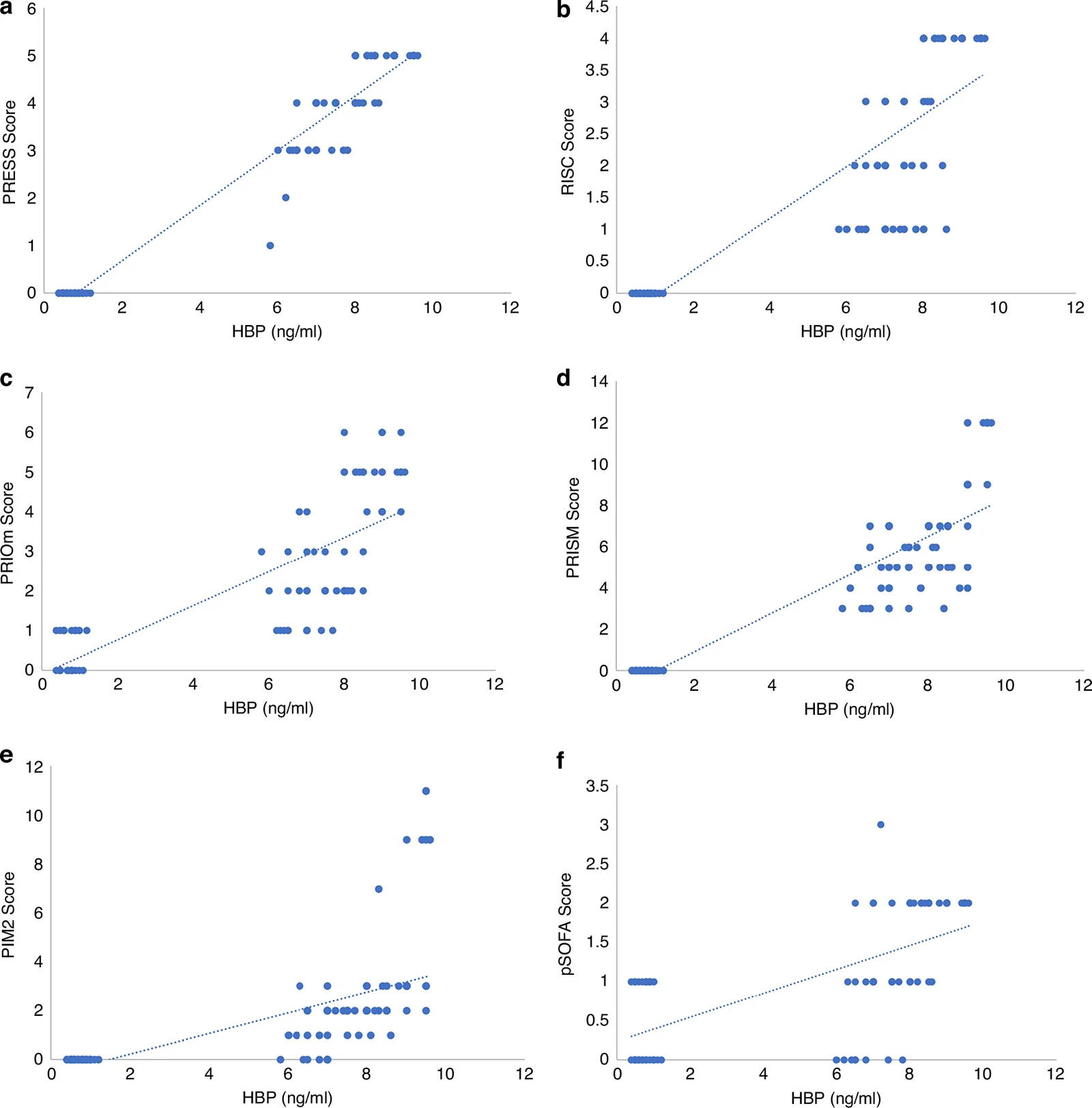
Correlation between HBP and clinical scores of severity. **A** Correlation between HBP and PRESS score. **B** Correlation between HBP and RISC score. **C** Correlation between HBP and PIROm score. **D** Correlation between HBP and PRISM score. **E** Correlation between HBP and PIM2 score. **F** Correlation between HBP and pSOFA score.

Correlation analysis demonstrated strong positive associations between HBP levels and various severity scoring systems, including the PRESS score (*r* = 0.944, *P* < 0.001), RISC score (*r* = 0.911, *P* = 0.001), PIROm score (*r* = 0.877, *P* = 0.001), PRISM score (*r* = 0.875, *P* = 0.001), PIM2 score (*r* = 0.845, *P* = 0.001), and pSOFA score (r = 0.739, *P* = 0.002) (Fig. 1).

Multivariate logistic regression identified several significant predictors of pneumonia severity, including WBC count, serum urea, albumin, carbon dioxide (CO₂), bicarbonate (HCO₃), HBP, and all severity scores assessed (PRESS, RISC, PIROm, PRISM, PIM2, and pSOFA) (Table 3).

**Table 3:** Multivariate logistic regression analysis for prediction of severity of pneumonia

Variable	aOR	95% CI	*P*
**Age (months)**	1.00	0.98–1.02	0.914
**Hb (g/dL)**	0.48	0.21–1.12	0.091
Platelets (*10^**9**^**/L)**	1.00	0.99–1.02	0.534
WBCs (*10^**9**^**/L)**	2.26	1.38–3.71	0.001*
**CRP (mg/L)**	1.01	0.93–1.05	0.997
**ALT (U/L)**	1.05	0.91–1.22	0.514
**AST (U/L)**	1.12	0.92–1.35	0.255
**Creatinine (mg/dL)**	0.02	0.00–3.93	0.324
**Urea (mg/dL)**	1.53	1.13–2.07	0.006*
**Albumin (g/dL)**	0.02	0.00–0.56	0.020*
**Na (mEq/L)**	0.16	0.01–2.23	0.172
**K (mEq/L)**	3.61	0.26–4.94	0.337
**Ca (mg/dL)**	0.99	0.98–1.00	0.159
**pH**	0.19	0.34–1.04	0.853
**CO2 (mmol/L)**	1.39	1.00–1.94	0.047*
**HCO3 (mEq/L)**	0.74	0.64–0.85	<0.001*
**HBP (ng/mL)**	3.23	1.72–6.06	<0.001*
**PRESS score**	2.70	1.21–2.82	<0.001*
**RISC score**	2.66	1.20–2.77	<0.001*
**PIROM score**	2.64	1.34–1.97	<0.001*
**PRISM score**	1.21	1.17–1.85	0.034*
**PIM2 score**	1.41	1.10–1.87	0.015*
**pSOFA score**	10.38	3.28–32.83	<0.001*

*Hb* hemoglobin, *WBCs* white blood cells, *CRP* C-reactive protein, *ALT* alanine aminotransferase, *AST* aspartate aminotransferase, *CO*_2_ carbon dioxide, *HCO*_3_ bicarbonate, *HBP* Heparin-binding protein, *PRESS* pediatric respiratory severity score, *RISC* respiratory index of severity in children, *PIROm* Predisposition, Insult, Response, and Organ dysfunction modified score, *PRISM* pediatric risk of mortality score, *PIM2* pediatric index of mortality-2, *pSOFA* pediatric sequential organ failure assessment, *aOR* adjusted odd ratio. *: statistically significant.

Statistically significant *p*-values are in bold.

**Fig. 2:**
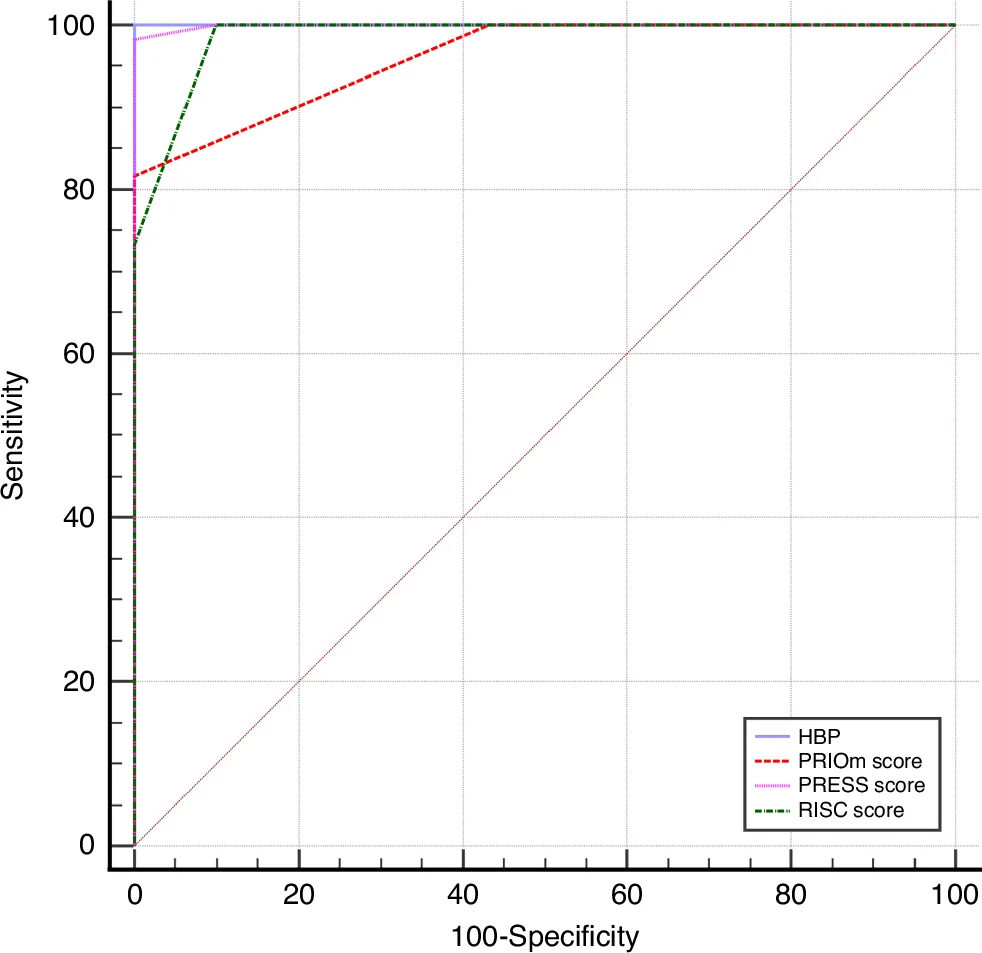
Receiver Operating Characteristic (ROC) curve analysis of HBP, PRESS, RISC, and PIROm scores for prediction of pneumonia severity. ROC curve analysis of HBP, PRESS, RISC, and PIROm scores for prediction of pneumonia severity.

Diagnostic performance analysis showed that HBP had an excellent predictive accuracy for severe pneumonia (AUC 0.962, sensitivity 100%, specificity 90% at a cutoff >1.2 ng/mL). Both PRESS and RISC achieved perfect discrimination (AUC 1.00, 100% sensitivity and specificity), while PIROm also demonstrated very good predictive value (AUC 0.960, sensitivity 81.7%, specificity 100% (Table 4 and Fig. 2).

**Table 4:** Diagnostic accuracy of HBP for prediction of severity of pneumonia

	Cutoff	Sensitivity	Specificity	PPV	NPV	AUC	*P* value
HBP (ng/ml)	>1.2	100	90.0	95.2	100	0.962	<0.001*
PRESS score	>0	100	100	100	100	1.00	<0.001*
RISC score	>0	100	100	100	100	1.00	<0.001*
PIROm score	>1	81.67	100	100	73.2	0.960	<0.001*

*HBP* Heparin-binding protein, *PRESS* pediatric respiratory severity score, *RISC* respiratory index of severity in children, *PIROm* Predisposition, Insult, Response, and Organ dysfunction modified score, *PPV* positive predictive value, *NPV* negative predictive value, *AUC* area under the curve, *: statistically significant.

Statistically significant *p*-values are in bold.

## Discussion

Various severity assessment tools have been developed to help clinicians predict outcomes such as mortality, ICU admission, and bacteremia, and are recommended by international guidelines to complement clinical judgment.^25^ Heparin-binding protein (HBP), a serine protease family member lacking conventional catalytic activity due to mutations in its triad, plays an important role in inflammation and host defense.^26,27^

It is primarily released from activated neutrophils in a calcium-dependent manner, triggered by microbial products and inflammatory mediators through pathways such as PI3K and p38 MAPK. Key stimuli include Staphylococcus aureus (viaPSMα4 /FPR2), Streptococcus Suis (viaLTB4/TLR4), and Streptococcus pyogenes (via M1protein/streptolysin O), while endothelin-1 also contributes to HBP elevation in sepsis.^28,29^

HBP is rapidly released upon neutrophil activation, preceding conventional inflammatory markers such as CRP and procalcitonin, thereby serving as an early indicator of infection.^30^ Its biological activity on the endothelium promotes vascular permeability and edema, key features of severe pneumonia.^31^ Consistent with previous studies, our findings confirm that elevated HBP levels are strongly associated with the severity and prognosis of pediatric community-acquired pneumonia (CAP), correlating with complicated disease and prolonged hospitalization.^32,33^ While traditionally regarded as a biomarker for bacterial infections in adults, we observed higher HBP levels particularly in Mycoplasma pneumoniae infections, likely reflecting cytokine-driven neutrophil recruitment.^34,35^ Overall, HBP levels appear to mirror CAP severity in children, and nomogram-based models incorporating HBP may help identify high-risk patients who would benefit from closer monitoring and timely intervention.^36,37^

The low mean weight and height observed in our cohort likely reflect the high prevalence of undernutrition in the population, consistent with national data. Undernutrition compromises immune defenses and increases susceptibility to severe respiratory infections; however, prior evidence shows no significant association between malnutrition indices and HBP concentrations, with HBP remaining an independent predictor of mortality.^38–40^

In our cohort, the need for mechanical ventilation was significantly higher among children with severe pneumonia compared to those with simple pneumonia, reflecting the risk of respiratory failure in severe cases.^41^ Similar findings have been reported by Bashir et al., who observed that children with CAP requiring intensive care had a median ICU stay of 8 days (IQR 5–14), whereas the overall hospitalized CAP cohort had a shorter median stay of 3.5 days (IQR 2–8) with no in-hospital deaths.^42^ Likewise, Loh et al. demonstrated that viral pneumonia was associated with greater illness severity and higher mechanical ventilation requirements compared to bacterial pneumonia in pediatric ICU patients.^43^

Large cohort studies have further shown that while respiratory failure and septic shock are major causes of early mortality in CAP, severity scores alone may not fully predict long-term outcomes, emphasizing the need for comprehensive management strategies.^44,45^

Children admitted to ICUs often present with severe conditions such as respiratory distress, sepsis, or multi-organ dysfunction, which contribute to elevated severity scores like PRESS and RISC.^46^ Validation studies have shown the prognostic value of these tools, with Abd El Megied et al. reporting significantly higher RISC scores in non-survivors compared to survivors.^47^ Similarly, Lu et al. found that prolonged invasive mechanical ventilation ( ≥ 96 hours) was associated with higher pSOFA scores, underscoring its correlation with pneumonia severity.^48^ Biomarker studies further highlight the diagnostic and prognostic potential of HBP, with Xiao et al. demonstrating its utility alongside conventional inflammatory markers in CAP patients.^49^ In our cohort, CRP levels were significantly higher in severe versus simple pneumonia, consistent with prior studies that linked elevated CRP to disease severity and worse outcomes.^42,50,51^

In our study, serum HBP levels were significantly higher in children with severe compared to simple pneumonia (*P* < 0.001), consistent with its role in increasing vascular permeability and edema.^52^ Prior studies support these findings: Li et al. reported nearly double HBP levels in severe or complicated CAP versus mild cases,^13^ while Liu et al. demonstrated markedly elevated HBP in severe sepsis compared with non-severe cases.^53^ Similarly, Xiao et al. observed significantly higher plasma HBP in CAP patients,^49^ and Huang et al. showed that children with severe CAP had median HBP levels exceeding the normal limit by more than eightfold.^11^ HBP has also been linked to disease severity scores, with Mishra et al. reporting correlations with RISC,^54^ Tang et al. with SOFA,^55^ and Tverring et al. demonstrating associations between higher daily HBP levels and greater cardiovascular dysfunction during septic shock.^56^

Multivariate logistic regression in our cohort identified white blood cell count, urea, albumin, CO₂, bicarbonate, HBP, and multiple severity scores (PRESS, RISC, PIROm, PRISM, PIM2, and pSOFA) as significant predictors of pneumonia severity. Consistent with this, Huang et al. reported that HBP outperformed traditional biomarkers for monitoring progression in severe pediatric CAP.^11^ In our study, HBP demonstrated excellent predictive accuracy, with an AUC of 0.962 at a cut-off >1.2 ng/mL, achieving 100% sensitivity and NPV, and 90% specificity with a PPV of 95.2% (*P* < 0.001). These results align with Li et al., who found HBP provided the strongest discrimination for severe or complicated CAP (adjusted OR = 3.11 at ≥60 ng/mL),^13^ and with Huang et al., who identified HBP as the best indicator of progression to severe sepsis (AUC 0.85, specificity 96.3%).^11^ Similarly, Mishra et al. demonstrated that an HBP cut-off of 41 ng/mL could predict fatal outcomes in children with respiratory distress.^54^

It is noteworthy that the absolute HBP concentrations observed in our study were lower than those reported in several previous clinical studies, where median values in healthy individuals are typically 6–12 ng/mL and thresholds for severe disease have been described in the range of 20–60 ng/mL. These differences are most likely attributable to methodological variation, as no international reference standard for HBP currently exists. Reported levels may vary depending on assay design (including antibody pairs and calibrator material), sample dilution protocols, and matrix effects. Importantly, in our cohort, values were internally consistent and demonstrated clear separation between high- and low-severity groups, with non-overlapping confidence intervals, thereby supporting the internal validity of our findings despite the lower absolute concentrations.

Severity scoring systems such as PRESS, RISC, and PIROm showed strong predictive performance for pneumonia severity in our cohort, with excellent AUC values and high sensitivity and specificity (*P* < 0.001). Validation studies support these findings, as Abd El Megied et al. demonstrated high sensitivity and specificity of the RISC score for mortality prediction in infants with CAP,^47^ while Pillai et al. reported that lower RISC cut-offs offer good sensitivity but limited specificity, with higher cut-offs improving specificity at the expense of sensitivity.^57^ Beyond RISC, El-Mashad et al. found that the SOFA score provided superior accuracy for predicting 30-day pediatric mortality (AUC 0.89) compared with PRISM and PIM2, and demonstrated moderate correlations with both scores.^58^

In our cohort, PRESS and RISC scores demonstrated perfect diagnostic accuracy, outperforming HBP, which nonetheless showed excellent predictive value (AUC 0.962). While validated clinical scores such as PRESS and RISC remain highly reliable for assessing severity and mortality risk in pediatric CAP.^47,58^ HBP provides the advantage of being a rapid, objective biomarker that reflects neutrophil activation and endothelial dysfunction. Rather than replacing clinical scores, HBP may serve as a complementary tool to enhance early risk stratification and support decision-making, particularly when bedside scoring is not feasible.

Our findings align with previous studies showing that elevated HBP levels correlate with CAP severity in children, including longer ICU stays, need for ventilation, and higher severity scores. These findings are summarized in Supplemental Table S1.

### Strengths and limitations

The strengths of this study include its prospective design, the simultaneous evaluation of pneumonia-specific and general pediatric critical illness scores, and complete data capture for all enrolled patients. However, several limitations should be acknowledged. HBP testing remains costly and limited to specialized laboratories, restricting its routine clinical use, particularly in low-resource settings. Only a single HBP measurement was obtained, which may not reflect its dynamic changes during illness. The exceptionally high AUCs for PRESS and RISC scores could indicate overfitting or sample-specific effects, and the absence of internal validation along with the relatively small sample size may limit generalizability. Finally, while 95% confidence intervals were included to improve interpretability, larger multicenter studies are needed to confirm these findings.

## Conclusion

Serum HBP levels are markedly elevated in critically ill children with severe CAP admitted to the PICU. Severe pneumonia is associated with prolonged hospital stays and increased need for, and duration of, mechanical ventilation. Elevated HBP strongly correlates with pneumonia severity and may outperform conventional biomarkers such as C-reactive protein (CRP) and white blood cells (WBCs) count in predicting disease progression, thus representing a valuable tool for early risk stratification and clinical management. Although elevated HBP was significantly associated with pneumonia severity, our analysis showed that PRESS and RISC scores had superior predictive performance, suggesting they may be more reliable tools for early risk stratification and guiding clinical management in pediatric CAP.

### Implications for practice and future research

HBP appears to be a valuable biomarker for early identification of children at risk of severe CAP. Its integration into clinical decision-making, either as a standalone marker or as part of a composite score, may improve early triage or guide the intensity of care. Future multicenter studies with larger cohorts are needed to validate these findings and establish standardized HBP cut-off values in pediatric populations. Incorporating newer versions as PRISM III/IV and PIM 3 may enhance predictive accuracy and comparability across centers. Additionally, evaluating HBP alongside updated scores in larger, multicenter cohorts would help confirm its utility relative to both pneumonia-specific and general critical illness tools.

## Supplementary information


DLR-AZU1-Hu
Supplementary HBP Table
supplementary references


## Data Availability

The datasets generated during and/or analyzed during the current study are available from the corresponding author on reasonable request.
