# Supplementary material for: Pediatric community-acquired pneumonia: predictive value of heparin-binding protein for severity assessment
Source: Pediatr Res. 2025 Dec 11;100(1):387–94. doi: 10.1038/s41390-025-04605-w (PMC13524798; doi:10.1038/s41390-025-04605-w)
Supplement: Supplementary file 1 [file 41390_2025_4605_MOESM1_ESM.pdf]

DEVELOP®

INSTRUCTION MANUAL

www.dldevelop.com

Human Azurocidin 1 (AZU1) ELISA Kit

Catalog No: DLR-AZU1-Hu

96 Tests

FOR RESEARCH USE ONLY; NOT FOR THERAPEUTIC OR DIAGNOSTIC APPLICATIONS!
PLEASE READ THROUGH ENTIRE procedure BEFORE BEGINNING!

INTENDED USE

The kit is a sandwich enzyme immunoassay for the in vitro quantitative measurement of AZU1 in human serum, plasma, tissue homogenates, cell lysates or other biological fluids.

REAGENTS AND MATERIALS PROVIDED

Reagents	Quantity	Reagents	Quantity
Pre-coated, ready to use 96-well strip plate	1	Plate sealer for 96 wells	2
Standard	2	Standard Diluent	1×20mL
Detection Solution A	1×12mL	TMB Substrate	1×9mL
Detection Solution B	1×12mL	Stop Solution	1×6mL
Wash Buffer (30 × concentrate)	1×20mL	Instruction manual	1

MATERIALS REQUIRED BUT NOT SUPPLIED

1. Microplate reader with 450 ± 10nm filter.
2. Precision single or multi-channel pipettes and disposable tips.
3. Eppendorf Tubes for diluting samples.
4. Deionized or distilled water.
5. Absorbent paper for blotting the microtiter plate.
6. Container for Wash Solution.

STORAGE OF THE KITS

1. For unopened kits: All the reagents should be kept at **4°C** upon receipt.
2. For opened kits: Once the kit is opened, the remaining reagents still need to be stored according to the above storage conditions. In addition, return the unused wells to the foil pouch containing the desiccant pack and reseal along entire edge of zip-seal.

Note:

For the expiration date of the kit, please refer to the label on the kit box. All components are stable until this expiration date.

It is highly recommended to use the remaining reagents within 1 month of opening.

SAMPLE COLLECTION AND STORAGE

- **Serum** - Use a serum separator tube and allow samples to clot for two hours at room temperature or overnight at 4°C before centrifugation for 20 minutes at approximately 1000×g. Assay freshly prepared serum immediately or store samples in aliquots at -20°C or -80°C for later use. Avoid repeated freeze/thaw cycles.

- **Plasma** - Collect plasma using EDTA or heparin as an anticoagulant. Centrifuge samples for 15 minutes at 1000×g at 2-8°C within 30 minutes of collection. Remove plasma and assay immediately or store samples in aliquots at -20°C or -80°C for later use. Avoid repeated freeze/thaw cycles.
- **Tissue homogenates** - The preparation of tissue homogenates will vary depending upon tissue type. For this assay, tissues should be rinsed in ice-cold PBS(0.01mol/L,pH 7.0-7.2) to remove excess blood thoroughly and weighed before homogenization. Mince the tissues to small pieces and homogenize them in 5-10 mL of PBS with a glass homogenizer on ice (Micro Tissue Grinders also work). The resulting suspension should be sonicated with an ultrasonic cell disrupter or subjected to two freeze-thaw cycles to further break the cell membranes. After that, the homogenates are centrifugated for 5 minutes at 5000×g. Remove the supernate and assay immediately or aliquot and store at ≤-20°C.
- **Cell Lysates** - Cells must be lysed before assaying according to the following directions.

1. Adherent cells should be detached with trypsin and then collected by centrifugation (suspension cells can be collected by centrifugation directly).
2. Wash cells three times in cold PBS.
3. Resuspend cells in PBS (1×) and subject the cells to ultrasonication 4 times (or Freeze cells at ≤-20°C. Thaw cells with gentle mixing. Repeat the freeze/thaw cycle 3 times.).
4. Centrifuge at 1500×g for 10 minutes at 2-8°C to remove cellular debris.

- **Other biological fluids** - Centrifuge samples for 20 minutes at 1000×g. Remove particulates and assay immediately or store samples in aliquots at -20°C or -80°C. Avoid repeated freeze/thaw cycles.

Note:

1. Samples to be used within 5 days may be stored at 4ºC, otherwise samples must be stored at -20ºC (≤1 month) or -80ºC (≤2 months) to avoid loss of bioactivity and/or contamination.
2. Sample hemolysis will influence the result, and hemolytic specimen can not be detected.
3. When performing the assay, bring samples to room temperature.

REAGENT PREPARATION

1. Bring all kit components and samples to room temperature (18-25ºC) before use.
2. **Standard** - Reconstitute the Standard with 1.0mL of Standard Diluent, keep for 10 minutes at room temperature, shake gently (not to foam). The concentration of the standard in the stock solution is 10ng/mL. Prepare 7 tubes containing 0.5mL Standard Diluent and use the diluted standard to produce a double dilution series according to the picture shown below. Mix each tube thoroughly before the next transfer. Prepare a dilution series with 7 points; for example: 10ng/mL, 5ng/mL, 2.5ng/mL, 1.25ng/mL, 0.625ng/mL, 0.312ng/mL, 0.156ng/mL, and the last EP tube with Standard Diluent is the blank at 0ng/mL.

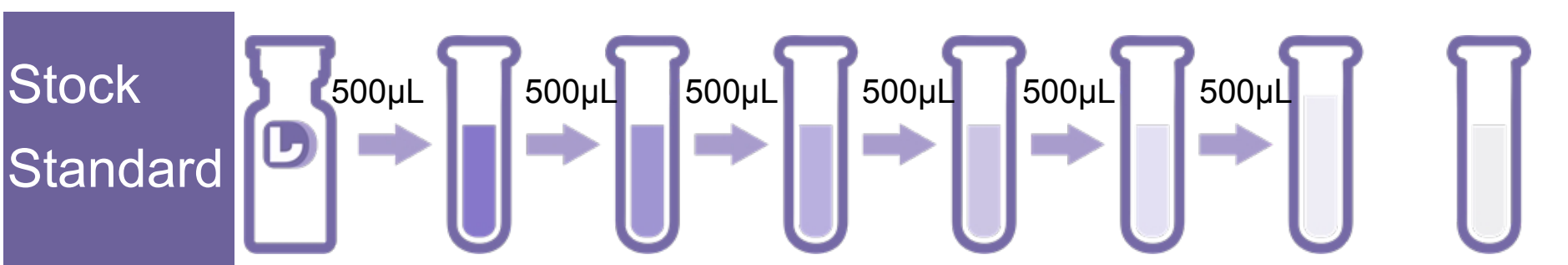

Tube	1	2	3	4	5	6	7	8
ng/mL	10	5	2.5	1.25	0.625	0.312	0.156	0

3. **Detection Solution A and Detection Solution B** - Detection

Solutions A and B are already at the correct concentrations and do not need to be diluted further.

4. **Wash Solution** - Dilute 20mL of Wash Solution concentrate (30×) with 580mL of deionized or distilled water to prepare 600mL of Wash Solution (1×).
5. **TMB substrate** - Aspirate the needed dosage of the solution with sterilized tips. Do not dump the residual solution back into the vial.

Note:

1. Do not perform a serial dilution directly in the wells.
2. Prepare standard within 15 minutes of performing the assay.
3. Carefully reconstitute Standards according to the instruction, avoid foaming and mix gently until the crystals are completely dissolved.
4. The reconstituted Standards can be **used only once**.
5. If crystals have formed in the Wash Solution concentrate (30×), warm to room temperature and mix gently until the crystals are completely dissolved.
6. Any contaminated water or container used during reagent preparation will influence the detection result.

SAMPLE PREPARATION

1. DL Sci & Tech Development Co.,Ltd. is only responsible for the kit itself, not for the samples consumed during the assay. The user should calculate the possible amount of the samples used in the whole test. Please reserve sufficient samples in advance.
2. Please predict the concentration before assaying. If values for these are not within the range of the standard curve, users must determine the optimal sample dilutions for their particular experiments. Samples should be diluted by 0.01mol/L PBS(pH=7.0-7.2).
3. If the samples are not indicated in the manual, a preliminary

experiment to determine the validity of the kit is necessary.
4. Tissue or cell extraction samples prepared using a chemical lysis buffer may cause unexpected ELISA results due to the impacts from certain chemicals.
5. Due to the possibility of mismatching between antigens from other origin and antibodies used in our kits (e.g., antibody targets conformational epitope rather than linear epitope), some native or recombinant proteins from other manufacturers may not be recognized by our products.
6. Samples from cell culture supernatant may not be detected by the kit due to influence from factors such as cell viability, cell number and/or sampling time.
7. Fresh samples that have not been stored for extended periods of time are recommended for the test. Otherwise, protein degradation and denaturalization may occur in those samples and give inaccurate or incorrect results.

ASSAY PROCEDURE

1. Determine wells for diluted standard, blank and sample. Prepare 7 wells for the standards, 1 well for blank. Add 100μL each of dilutions of standard (read Reagent Preparation), blank, and samples into the appropriate wells. Cover with the Plate sealer. Incubate for 90 minutes at 37°C.
2. Remove the liquid from each well, do not wash.
3. Add 100μL of Detection Solution A to each well. Incubate for 45 minutes at 37°C after covering it with the Plate sealer.
4. Aspirate the solution and wash with 300μL of 1× Wash Solution to each well using a squirt bottle, multi-channel pipette, manifold

dispenser or auto-washer, and let it sit for 1-2 minutes. Remove the remaining liquid from all wells completely by tapping the plate onto absorbent paper. Wash thoroughly 3 times. After the last wash, remove any remaining Wash Buffer by aspirating or decanting. Invert the plate and blot it against absorbent paper.

5. Add 100μL of Detection Solution B to each well. Incubate for 45 minutes at 37°C after covering it with the Plate sealer.
6. Repeat the aspiration/wash process for a total of 5 times as conducted in step 4.
7. Add 90μL of Substrate Solution to each well. Cover with a new Plate sealer. Incubate for 15-25 minutes at 37°C (Do not exceed 30 minutes). Protect from light. The liquid will turn blue with the addition of the Substrate Solution.
8. Add 50μL of Stop Solution to each well. The liquid will turn yellow with the addition of the Stop solution. Mix the liquid by tapping the side of the plate. If the color change does not appear uniform, gently tap the plate to ensure thorough mixing.
9. Remove any drops of water and fingerprints on the bottom of the plate and confirm there are no bubbles on the surface of the liquid. Run the microplate reader and take measurements at 450nm immediately.

Note:

1. **Assay preparation:** Keep appropriate numbers of wells for each experiment and remove extra wells from microplate. Remaining wells should be resealed and stored at 4°C.
2. **Samples or reagents addition: Please use the freshly prepared Standard.** Carefully add samples to wells and mix gently to avoid foaming. Do not touch the well walls. For each step in the procedure, total dispensing time for addition of reagents or samples to the assay plate should not exceed 10 minutes. This will ensure equal elapsed time for each pipetting step, without interruption. Duplication of all

standards and specimens, although not required, is recommended. To avoid cross-contamination, change pipette tips between additions of standards, samples, and reagents. In addition, use separated reservoirs for each reagent.

3. **Incubation:** To ensure accurate results, proper adhesion of plate sealers during incubation steps is necessary. Do not allow wells to sit uncovered for extended periods in between incubation steps. Once reagents are added to the well strips, DO NOT let the strips dry at any time during the assay. Incubation time and temperature must be controlled.
4. **Washing:** The wash procedure is critical. Complete removal of liquid at each step is essential for good performance. After the last wash, remove any remaining Wash Solution by aspirating or decanting, and remove any drops of water or fingerprints on the bottom of the plate. Insufficient washing will result in poor precision and false elevated absorbance reading.
5. **Controlling of reaction time:** Observe the change of color after adding **TMB Substrate** (e.g. observation once every 10 minutes), if the color is too deep, add Stop Solution in advance to avoid excessively strong reaction which will result in an inaccurate absorbance reading.
6. **TMB Substrate** is easily contaminated. Please protect it from light.
7. The environment humidity may have an effect on the results obtained from the kit. If the humidity in your facility is less than 60%, using a humidifier is recommended.

TEST PRINCIPLE

The microtiter plate provided in this kit has been pre-coated with an antibody specific to AZU1. Standards or samples are then added to the

appropriate microtiter plate wells with a biotin-conjugated antibody preparation specific to AZU1. Next, Avidin conjugated to Horseradish Peroxidase (HRP) is added to each microplate well and incubated. After TMB substrate solution is added, only those wells that contain AZU1, biotin-conjugated antibody and enzyme-conjugated Avidin will exhibit a change in color. The enzyme-substrate reaction is terminated by the addition of sulphuric acid solution and the color change is measured spectrophotometrically at a wavelength of 450nm ± 10nm. The concentration of AZU1 in the samples is then determined by comparing the O.D. of the samples to the standard curve.

CALCULATION OF RESULTS

Average the duplicate readings for each standard, control and sample, then subtract the average zero standard optical density. Construct a standard curve by plotting the mean O.D. and concentration for each standard and draw a best fit curve through the points on the graph or create a standard curve on log-log graph paper with AZU1 concentration on the y-axis and absorbance on the x-axis. Using ploting software, (for instance, curve expert 1.30), is also recommended. If samples have been diluted, the concentration read from the standard curve must be multiplied by the dilution factor.

TYPICAL DATA

In order to make the calculation easier, we plot the O.D. value of the standard (X-axis) against the known concentration of the standard (Y-axis), although concentration is the independent variable and O.D. value is the dependent variable. However, the O.D. values of the standard curve may vary according to the conditions of assay performance (e.g. operator,

pipetting technique, washing technique or temperature effects), plotting the log of the data to establish a standard curve for each test is recommended. The typical standard curve below is provided for reference only.

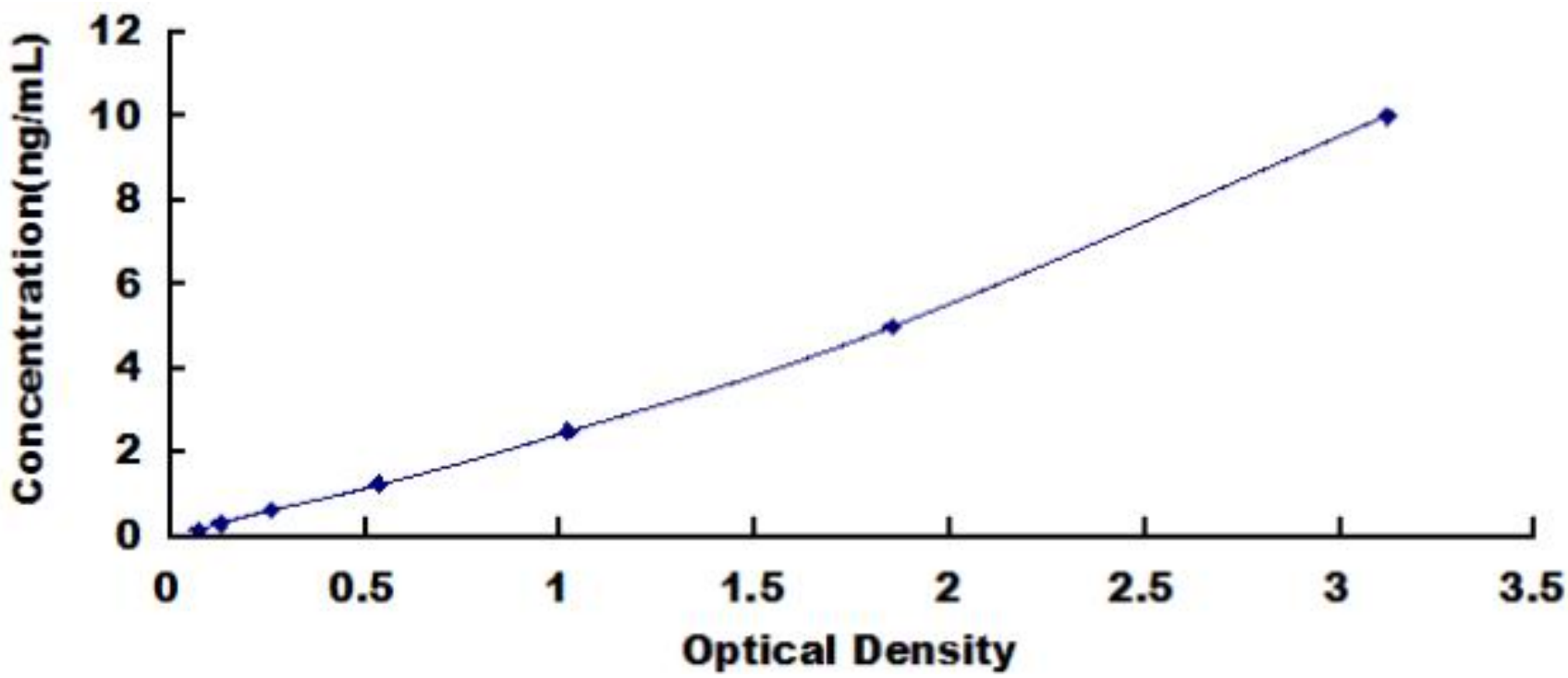

Typical Standard Curve for Human AZU1 ELISA.

DETECTION RANGE

0.156-10ng/mL. The standard curve concentrations used for the ELISA's were 10ng/mL, 5ng/mL, 2.5ng/mL, 1.25ng/mL, 0.625ng/mL, 0.312ng/mL, 0.156ng/mL.

SENSITIVITY

The minimum detectable dose of AZU1 is typically less than 0.051ng/mL. The sensitivity of this assay, or Lower Limit of Detection (LLD) was defined as the lowest protein concentration that could be differentiated from zero. It was determined by adding two standard deviations to the mean optical density value of twenty zero standard replicates and calculating the corresponding concentration.

SPECIFICITY

This assay has high sensitivity and excellent specificity for detection of AZU1.

No significant cross-reactivity or interference between AZU1 and analogues was observed.

Note:

Limited by current skills and knowledge, it is impossible to perform all possible cross-reactivity detection tests between AZU1 and all analogues, therefore, cross reactivity may still exist.

PRECISION

Intra-assay Precision (Precision within an assay): 3 samples with low, middle and high level AZU1 were tested 20 times on one plate, respectively.

Inter-assay Precision (Precision between assays): 3 samples with low, middle and high level AZU1 were tested on 3 different plates, 8 replicates in each plate.

CV(%) = SD/meanX100

Intra-Assay: CV<10%

Inter-Assay: CV<12%

STABILITY

The stability of ELISA kit is determined by the loss rate of activity. The loss rate of this kit is less than 5% within the expiration date under appropriate storage conditions.

Note:

To minimize unnecessary influences on the performance, operation

procedures and lab conditions, especially room temperature, air humidity, and incubator temperatures should be strictly regulated. It is also strongly suggested that the whole assay is performed by the same experimenter from the beginning to the end.

ASSAY PROCEDURE SUMMARY

1. Prepare all reagents, samples and standards;
2. Add 100μL standard or sample to each well. Incubate 90 minutes at 37°C;
3. Aspirate and add 100μL Detection Solution A. Incubate 45 minutes at 37°C;
4. Aspirate and wash 3 times;
5. Add 100μL Detection Solution B. Incubate 45 minutes at 37°C;
6. Aspirate and wash 5 times;
7. Add 90μL Substrate Solution. Incubate 15-25 minutes at 37°C;
8. Add 50μL Stop Solution. Read at 450nm immediately.

IMPORTANT NOTES

1. Limited by the current conditions and scientific technology, it is impossible to conduct comprehensive identification and analysis tests on the raw materials provided by suppliers. As a result, it is possible there are some qualitative and/or technical risks.
2. The final experimental results will be closely related to the validity of the products, operation skills of the end users and the experimental environments. Please make sure that sufficient samples are available to obtain accurate results.
3. Kits from different batches may be a little different in detection range,

sensitivity and color developing time. Please perform the experiment exactly according to the instruction manual included in your kit. Electronic ones on our website are for reference only.

4. Do not mix or substitute reagents from one kit lot to another. Use only the reagents supplied by manufacturer.
5. Protect all reagents from strong light during storage and incubation. All bottle caps of reagents should be closed tightly to prevent evaporation of liquids and contamination by microorganisms.
6. There may be a foggy substance in the wells when the plate is opened at the first time. It will not have any effect on the final assay results. Do not remove microtiter plate from the storage bag until needed.
7. Incorrect procedures during reagent preparation and loading, as well as incorrect parameter setting for the plate reader may lead to incorrect results. A microplate plate reader with a bandwidth of 10nm or less and an optical density range of 0-3 O.D. or greater at 450 ± 10nm wavelength is acceptable for use in absorbance measurement. Please read the instruction carefully and adjust the instrument prior to the experiment.
8. Even the same experimenter may get different results from two separate experiments. In order to get better reproducible results, the operation of every step in the assay should be controlled. Furthermore, a preliminary experiment before the general assay for each batch is recommended.
9. Each kit has undergone several rigorous quality control tests. However, results from end users might be inconsistent with our in-house data due to some unexpected transportation conditions or different lab equipment. Intra-assay variance among kits from different batches could arise from the above factors as well.
10. Kits from different manufacturers with the same item might produce different results, since we have not compared our products with other

DEVELOP

manufacturers.

11. The standard in this kit, as well as the antigens used in antibody preparation are typically recombinant proteins. Differently expressed sequences, expression systems, and/or purification methods can be used in the preparation of recombinant proteins. There is also the possibility of differences in the screening technique of antibodies and antibody pairs in our kits. As a result, we cannot guarantee that our kit will be able to detect recombinant proteins produced by other companies. We do NOT recommend using our ELISA kits for the detection of other recombinant proteins.
12. Validity period: 12 months.
13. The instruction manual also works with the 48T kit, but all reagents in the 48T kit are reduced by half.

PRECAUTION

The Stop Solution suggested for use with this kit is an acid solution. Wear eye, hand, face, and clothing protection when using this reagent.
