# Supplementary material for: Pediatric community-acquired pneumonia: predictive value of heparin-binding protein for severity assessment
Source: Pediatr Res. 2025 Dec 11;100(1):387–94. doi: 10.1038/s41390-025-04605-w (PMC13524798; doi:10.1038/s41390-025-04605-w)
Supplement: Supplementary file 2 [file 41390_2025_4605_MOESM2_ESM.pdf]

Supplementary Table (1): Summary of key studies evaluating HBP in Pediatric Pneumonia and Critical Illness

Study (Year)	Country	Population (n)	Key Findings	Notable Results
**Li et al. (2018)**	China	120 pediatric CAP	Higher HBP in severe/complicated CAP vs. mild	Median HBP: 43.0 vs. 24.3 ng/mL ($p < 0.05$)
**Huang et al. (2019)**	China	86 critically ill children with severe CAP	HBP elevated in severe cases	Median HBP: 98.1 ng/mL (>8× normal)
**Mishra et al. (2020)**	India	150 children with respiratory distress	HBP correlated with RISC score and poor outcome	$r = 0.11$, $p < 0.0001$; Cut-off: 41 ng/mL
**Bashir et al. (2022)**	Pakistan	200 pediatric CAP (ICU cohort)	Severity linked with LOS and biomarkers	ICU LOS 8 days; CRP ↑ with higher HBP
**Xiao et al. (2021)**	China	95 CAP patients (ED cohort)	Elevated HBP in CAP vs. controls	$p < 0.001$
**Liu et al. (2020)**	China (multicenter)	240 pediatric PICU patients with sepsis	HBP as diagnostic/prognostic marker	Median HBP: 170.5 vs. 74.1 ng/mL ($p < 0.001$)
**Tang et al. (2017)**	China	110 critically ill septic patients	HBP positively correlated with SOFA	$p < 0.05$
**Tverring et al. (2020)**	Sweden	120 septic shock patients	High HBP linked to cardiovascular dysfunction	Higher HBP = ↑ SOFA over first 3 ICU days

HBP: Heparin-Binding Protein, CAP: Community-Acquired Pneumonia, LOS: length of stay, ED: emergency department, PICU: Pediatric Intensive Care Unit, SOFA: Sequential Organ Failure Assessment, RISC: Respiratory Index of Severity in Children, r: Spearman correlation coefficient, ↑: increased.
