# Supplementary material for: Pediatric community-acquired pneumonia: predictive value of heparin-binding protein for severity assessment
Source: Pediatr Res. 2025 Dec 11;100(1):387–94. doi: 10.1038/s41390-025-04605-w (PMC13524798; doi:10.1038/s41390-025-04605-w)
Supplement: Supplementary file 3 [file 41390_2025_4605_MOESM3_ESM.pdf]

Supplementary References:

1. Li S, Zhang Y, Chen J, Xu J, Zhao L, Liu P. Heparin-binding protein: a prognostic biomarker associated with severe or complicated community-acquired pneumonia in children. J Inflamm Res. 2023; 16:321–331.

2. Huang C, Zhang C, Zhang J, Zhang L, Mo Y, Mo L. Heparin-binding protein in critically ill children with severe community-acquired pneumonia. Front Pediatr. 2021; 9:759535.

3. Mishra H, Nanjappa S, Ssekitooleko J, Lubega I, Cummings MJ, Moore CC. Heparin-binding protein stratifies mortality risk among Ugandan children hospitalized with respiratory distress. Open Forum Infect Dis. 2024; 11:ofae030.

4. Bashir A, Khan R, Thompson S, Caceres M. A retrospective observational study of biomarker levels and severity assessment in pediatric community-acquired pneumonia. Medicine (Baltimore). 2022; 101(24):e30010.

5. Xiao X, Hong Y, Wang S, Ma M, Xu Z. Diagnostic value of plasma heparin-binding protein and the heparin-binding protein-to-albumin ratio in patients with community-acquired pneumonia: a retrospective study. BMC Infect Dis. 2023; 23:777.

6. Liu P, et al. Heparin-binding protein as a biomarker of severe sepsis in the pediatric intensive care unit: a multicenter, prospective study. Clin Chim Acta. 2023;539:26–33.

7. Tang J, Yuan H, Wu YL, Fu S, Pan XY. The predictive value of heparin-binding protein and D-dimer in patients with sepsis. Int J Gen Med. 2023; 16:2295–2303.

8. Tverring J, Hedberg P, Lipcsey M, Larsson A, Sjölin J, Ljungström L. Heparin-binding protein kinetics in septic shock. Crit Care. 2020; 24:93.
